# Recombination and Population Mosaic of a Multifunctional Viral Gene, Adeno-Associated Virus *cap*

**DOI:** 10.1371/journal.pone.0001634

**Published:** 2008-02-20

**Authors:** Yasuhiro Takeuchi, Richard Myers, Olivier Danos

**Affiliations:** 1 Medical Reserach Council (MRC)/UCL Centre for Medical Molecular Virology, Division of Infection and Immunology, University College London, London, United Kingdom; 2 Centre for Infections, Health Protection Agency, London, United Kingdom; 3 Institut National de la Santé et de la Recherche Médicale (INSERM), U781, Institut Fédératif de Recherche Necker Enfants Malades (IFR 94), Hôpital Necker Enfants Malades, Paris, France; Institute of Human Virology, United States of America

## Abstract

Homologous recombination is a dominant force in evolution and results in genetic mosaics. To detect evidence of recombination events and assess the biological significance of genetic mosaics, genome sequences for various viral populations of reasonably large size are now available in the GenBank. We studied a multi-functional viral gene, the adeno-associated virus (AAV) *cap* gene, which codes for three capsid proteins, VP1, VP2 and VP3. VP1-3 share a common C-terminal domain corresponding to VP3, which forms the viral core structure, while the VP1 unique N-terminal part contains an enzymatic domain with phospholipase A2 activity. Our recombinant detection program (RecI) revealed five novel recombination events, four of which have their cross-over points in the N-terminal, VP1 and VP2 unique region. Comparison of phylogenetic trees for different *cap* gene regions confirmed discordant phylogenies for the recombinant sequences. Furthermore, differences in the phylogenetic tree structures for the VP1 unique (VP1u) region and the rest of *cap* highlighted the mosaic nature of *cap* gene in the AAV population: two dominant forms of VP1u sequences were identified and these forms are linked to diverse sequences in the rest of *cap* gene. This observation together with the finding of frequent recombination in the VP1 and 2 unique regions suggests that this region is a recombination hot spot. Recombination events in this region preserve protein blocks of distinctive functions and contribute to convergence in VP1u and divergence of the rest of *cap*. Additionally the possible biological significance of two dominant VP1u forms is inferred.

## Introduction

**Figure 1:**
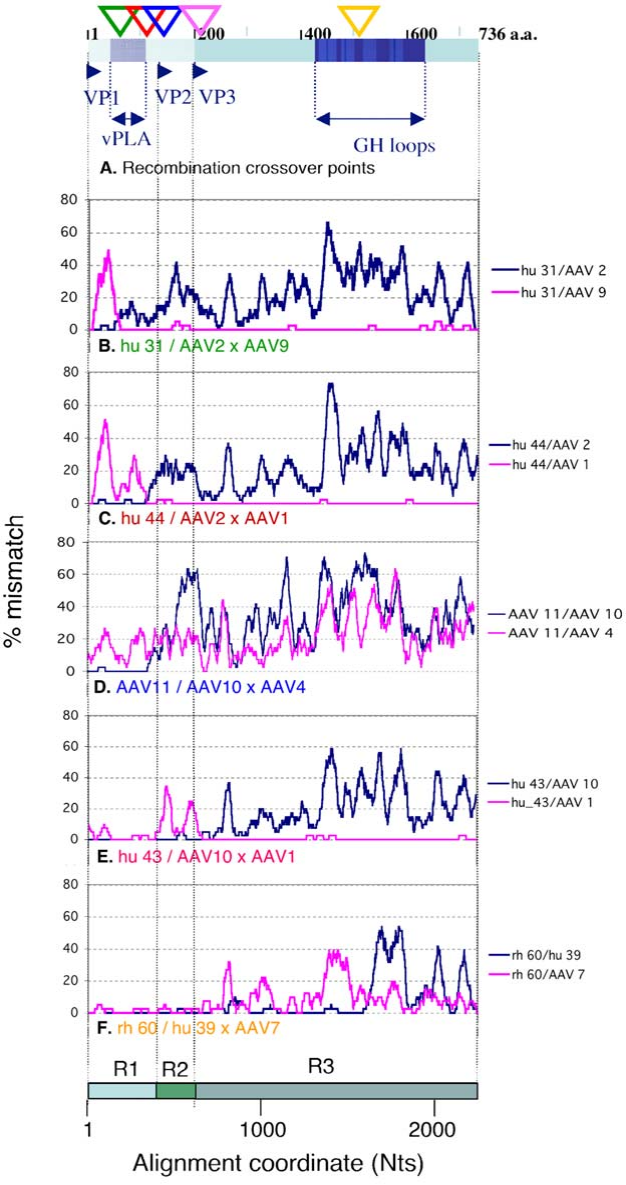
Profiles and cross-over points of novel recombination in the AAV cap gene. A. Schematic drawing of the *cap* gene products (VP1-3) with five recombination cross-over points inferred by LOHA analyses below (triangles colour-coordinated with Panels B-F) is shown. Viral phospholipase A and antigenic GH loop domains are indicated. B–F. Recombination profiles for representative combinations of 3 sequences (recombinant/parent 1×parent 2) were visualised by the LOHA analysis. Percent mismatch across a sliding window of 41 Nt positions between recombinant/parent pairs were plotted against the nucleotide positions along the multiple alignment with a step interval of 1 position. The x-axis also indicates three regions (R1-3) separately analysed in the following studies (see Fig 2 and 3).

Parvoviruses are eukaryotic viruses that infect a wide variety of hosts from insects to human and non-human primates. They are composed of a small icosahedral capsid, 22 to 24 nm in diameter into which is packaged a single stranded DNA genome of less than 5.6 kilobases with terminal palindromic sequences. In recent years, studies of the carnivore parvovirus subgroup have allowed the observation of cross species transmission between cat and dog populations. This host range shift was due to a high mutation rate in the capsid protein gene that enabled rapid adaptation to a new cell surface receptor [1], [2]. Host range variation has also been studied among Adeno-Associated Viruses (AAV), which are helper-dependent Parvoviruses (dependoviruses) with great potential as gene transfer vectors [3]. The capsid of AAV is formed by three proteins, VP1, VP2 and VP3 which are encoded by the same *cap* gene and differ in their N-terminal domain (Fig 1A). The common C-terminal domain corresponds to VP3 and folds into capsomer units that display a typical beta-barrel structure [4] with solvent exposed loops of variable amino-acids sequences. These variable regions of VP3 form the structural elements that recognize cell surface receptors. About one tenth of the capsomers are VP1 or VP2 and display additional N-terminal domains.

Beyond receptor recognition, AAV capsid proteins have been involved in the complex processes of viral entry and trafficking to the nucleus. The additional domains of VP1 and VP2 are buried inside the capsid in the native virions and become surface exposed during trafficking through the endosomal system [5]–[7]. This conformational change reveals basic amino acids which have been found to enhance nuclear targeting [7], [8]. It also activates a Phospholipase A2 (PLA2) which is encoded by the VP1 unique region (VP1u) and is conserved in all parvoviruses [9]. PLA2 activity is essential for endosomal escape and entry of the viral DNA into the nucleus [7].

Over one hundred different AAV isolates have been characterized in humans and non-human primates. They display a high degree of sequence variation in the *cap* gene regions encoding exposed loops and the receptor binding domain [10]. This variability allows AAV to escape the host immune responses and to adapt to different receptors. Phylogenetic studies of the cap sequences indicate that the numerous AAV isolates have probably evolved from a single parent of avian origin and are now distributed in clades, A-F [10]. Initial sequence comparisons have suggested that certain isolates could be the result of recombination between divergent parental genomes: for example Clade C. a predominantly human clade, has been proposed to be a hybrid of two AAV serotypes, AAV2 (Clade B) and AAV3 [10]–[13]. It is therefore possible that the structurally and functionally distinct regions of the cap gene have different evolutionary histories. In this study we reveal the mosaic genetic nature of the *cap* gene in individual virus isolates as well as in the AAV population for which sequence data are available. We discuss the relationship between such genetic mosaicism and the *cap* gene functions, as well as the implications for AAV biology and epidemiology.

## Results

**Figure 2:**
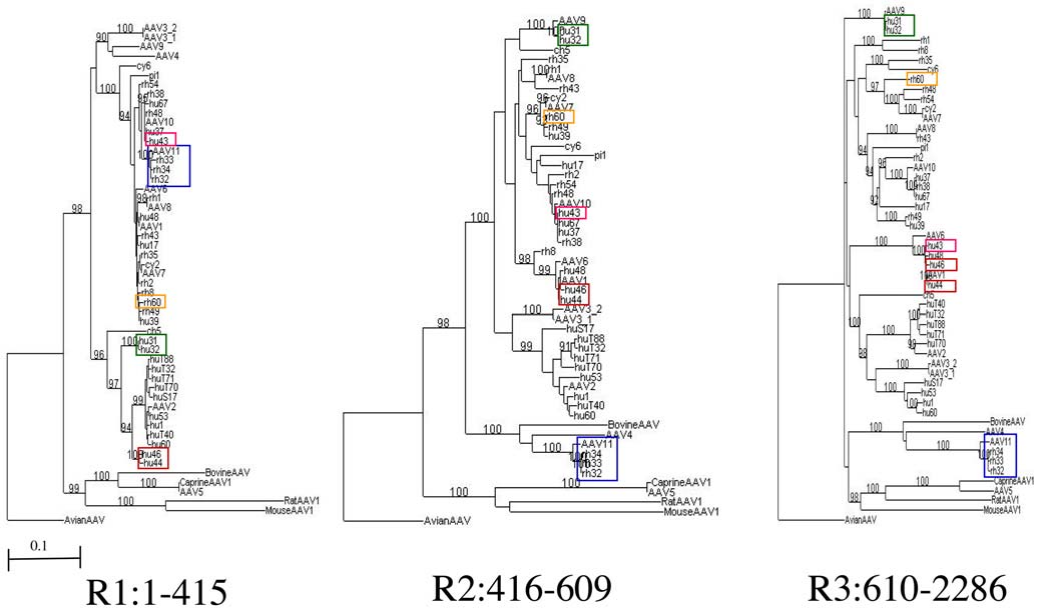
Local phylogenetic trees between 53 AAV cap sequences. Neighbour-joining trees were produced based on the nucleotide alignment for 3 regions: R1, VP1 unique; R2, between VP2 and VP3 N-termini; R3, VP3 coding region shared by all VP 1-3 (see Fig 1). Phylogenetic discordance between the three regions was observed for recombinant sequences. Tree structure difference between regions, particularly R1 versus R3, demonstrates the mosaic nature of the *cap* gene. Fifty-three sequences were selected by excluding sequences of non-mammalian origin (except an avian AAV used as the root), sequences derived from Tulane/F953 monkey and a number of ‘redundant’ sequences as indicated in Table S1. Bootstrap scores greater than 90 % are shown. Recombinant sequences depicted in Fig 1B–F are colour-boxed. A branch length scale common for all three trees is shown.

Available AAV *cap* nucleotide sequences, including those of human, mammalian, avian and reptilian origins, were retrieved from Genbank and aligned (n = 156). This alignment was used for recombinant sequence screening (Fig 1) and generation of phylogenetic trees for different regions of the *cap* gene (Fig 2) as described in Materials and Methods. Representative recombinant and parent relationships for the five recombination events were analysed using LOHA [14] (Fig 1, Panels B–F). For example, Panel B shows pair-wise comparisons generated by plotting the percentage of mismatches between hu 31 and AAV2 (blue) and between hu 31 and AAV9 (pink) (Y axis) for 41 base pair (bp) sliding windows along the alignment (X-axis). The first 150 bp of hu 31 are almost identical to those of AAV2 and different from AAV9, while this relationship is reversed in the rest of the genome which is nearly identical to AAV9 and different from AAV2. We infer from this result that hu 31 is a recombinant containing AAV2 and AAV9 like sequences in mosaic. While Panels B, C and E show similar mosaic patterns in *cap*, involving two different parent viruses, panels D and F show a more complex picture, with segments of low sequence homology for which no close parent can be identified. Three other sequences, rh 32–34, were closely related to AAV11 and showed similar LOHA profiles, while hu 31 and hu 44 shared the recombinant features of hu32 and hu46 respectively (data not shown, see Fig 2). It is likely that these sequences with the same recombination patterns have resulted from single recombination events. Recombination screening detected five recombination cross-over points the positions of which are indicated on the cap gene in Fig 1A. Our recombination screening also detected the previously reported recombination events observed in a single rhesus macaque (Tulane/F953) isolate [12]. Unlike the Tulane/F953 derived sequences, some of these novel recombinants, if not all, are likely to represent established isolates/strains and represent recombination events that resulted in viable, naturally selected recombinant forms of AAV. Interestingly, the five cross over points are not evenly distributed along the cap gene, as four of them occur in the N-terminus proximal part of cap that encode unique VP1 and VP2 sequences, suggesting that this part may be a region of frequent recombination.

The generation of local phylogenetic trees confirmed the finding of the recombinant and mosaic nature of the *cap* gene. Figure 2 shows local trees for the alignments of selected sequences in VP1 unique region (R1), the region between VP2 and VP3 initiation (R2) and the C-terminal region shared by all (R3). Sequences of non-mammalian origin, Tulane/F953 derived sequences and a number of ‘redundant’ sequences of human origin were excluded in this analysis. Phylogenetic discordance between the three regions was observed, such that recombinant sequences belonged to one cluster in a tree but moved to another cluster separated from the former cluster with high probability (high bootstrap score) in another tree. Such sequence jumps between branches are demonstrated for sequences, hu 44 and hu 46 (red in R1 and R2 trees), AAV11 and rh 32–34 (blue in R1 and R2), and hu 43 (pink in R2 and R3). The association of hu31 and hu32 (green) with AAV9 is evident in R2 and R3, but not R1, while the phylogenetic discordance of rh 60 (yellow) within R3 is not obvious but reflected by its long terminal branch in R3 tree (Fig 2).

Figure 2 also shows a striking tree structure difference between regions, particularly R1 versus R3, demonstrating the mosaic nature of the *cap* gene within these AAV sequences. The R1 tree, corresponding to the VP1 unique/PLA coding region has few long branches with tightly populated clusters, whereas the R3 tree corresponding to the capsid structure has many more long branches with less populated clusters. This observation can be interpreted simply as a consequence of different levels of conservation between these regions; R1 is a homologous, conserved region and R3 a variable antigenic region. However the conservation of R1 is not uniform within the population, as sequences separated by long branches appear to be substantially divergent in both R1 and R3: for example the mismatch between AAV1 and AAV2 in R1 and R3 is 16.8% and 22.5%, respectively. It is possible that R1 can take only few divergent forms due to functional restrictions, while varying forms of R3 are tolerated and/or necessary.

**Figure 3:**
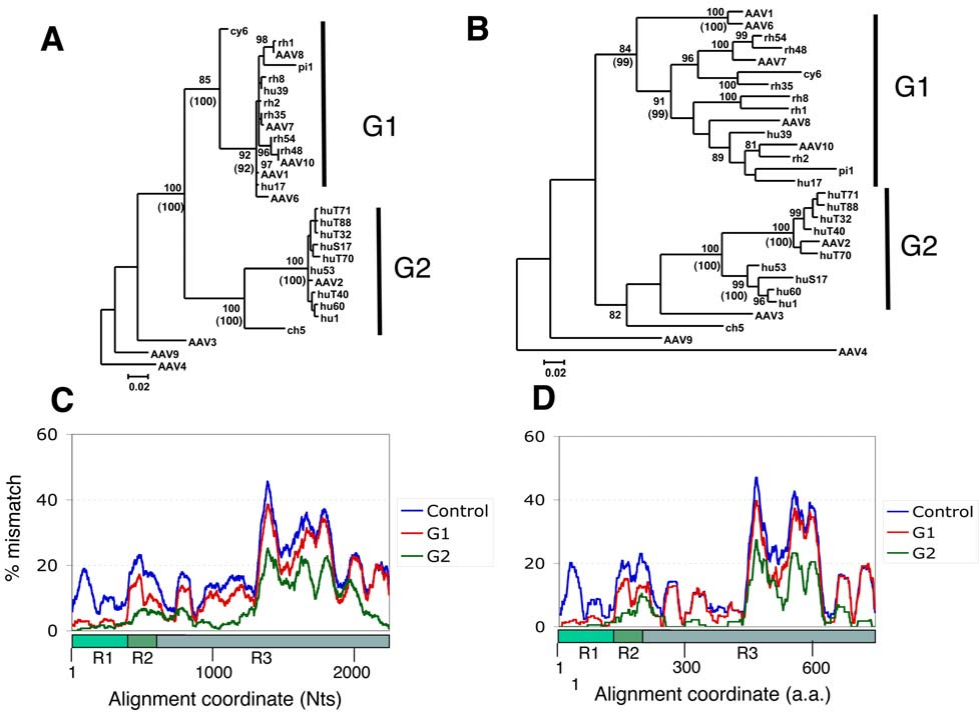
Identification of two sequence groups (G1 and G2) tightly clustered in the VP1 unique region. A. A Phyml maximum likelihood (ML) tree for R1 region was generated for a dataset of 29 sequences (see selection in Table S1). AAV4 was used as the outgroup and the bootstrap scores greater than 80 % are shown. Tightly clustered sequence groups, G1 of 15 sequences and G2 of 10 sequences, were identified and indicated. A similar tree was obtained using neighbour-joining method and its bootstrap scores that support G1 and G2 clustering are shown in parentheses. B. An ML tree for the full-length cap was obtained and shown in the same way as the R1 tree (Panel A). C. Average group LOHA profiles based on the nucleotide alignment are shown. Percent mismatch scores across a sliding window of 101 Nt positions for all possible pairs between all 29 sequences (Control), 15 G1 sequences (G1) and 10 G2 sequences (G2) were averaged and plotted against the nucleotide positions. D. Similar average LOHA profiles with 33 a.a. window size were obtained based on the amino acid sequence alignment.

To further define this regional difference in the phylogenetic relationship within the AAV population, we analysed a data set selected by excluding apparent recombinant sequences and redundant sequences. For this we selected one representative of each cluster of sequences with more than 98.5 % homology over the full *cap* gene. Four sequences, AAV3, AAV4, AAV9 and ch 5, were included as outsiders in this analysis. Figure 3A and B shows the resulting trees for R1 and the full *cap* gene respectively. In these trees, it is apparent that there are two dominant forms of R1. Sequences containing these two dominant forms were classified into Group 1 (G1) and 2 (G2), represented by AAV1 and AAV2, respectively (Fig 3A, G1 of 15 members and G2 of 10 members). R1 sequences in both G1 and G2 form tight clusters with high bootstrap values and for the most part low intra-cluster branch-lengths (less than 0.02 substitutions) (Fig 3A). The cluster structure of G1 was not absolutely conserved due to variation of the placement of sequences cy 6 and pi 1 using both maximal likelihood (ML) and neighbour joining (NJ) methodologies. The outlying sequence (ch 5) was shown to cluster robustly (bootstrap values 100, ML and NJ) with G2 sequences but with a far greater branch length than other members of this cluster. ML and NJ analysis of the full cap gene illustrated that the sequences within G1 and G2 remain clustered, but show substantial diversity depicted by long intra-cluster branches (Fig 3B).

To reveal where the members of G1 and G2 are homologous within the group and where they divert, pair-wise LOHA analyses were performed between 29 selected sequences (total 406 possible pairs). Figure 3C shows the averaged LOHA plots (101 bp sliding window) for the DNA alignment of all 406 sequence pairs, for 105 sequence pairs of G1 and 45 G2 sequence pairs. The 406 sequence pair average was included as a control indicating the diversity within the 29 sequence population along the alignment. While this graph shows the greatest variability in the middle part of VP3 (ca. 1300–1900 Nts), all 101 bp windows including VP1 unique region (R1, 1–415 Nts) have more than 5% mismatches in the average DNA LOHA for 406 sequence pair (Fig 3C). In contrast, the average for 105 G1 pairs showed low scores of less than 5 % at 1–403 positions, while the rest of the graph possesses a similar pattern to the control, with a sharp increase around the position 400. Because the window at position 400 covers bp positions that extend to bp 450, the region represented by 1–400 in this analysis roughly corresponds to R1. The ratio of these two plots (G1/Control) scored less than 30 % at positions 4–390, but most positions in the rest were 40–100 % (data not shown), demonstrating the peculiar, mosaic diversity pattern of the G1 sequences. The average for G2 pairs also shows a similar pattern to G1 average, as positions ca. 1–400 have low scores with an increase downstream, while this population has another region of low scores (bp 900–1200).

Average plots were also obtained for protein alignments of 29 predicted Cap amino acid sequences, using a 33 a.a. window (Fig. 3D). A pattern similar to the one described above for DNA LOHA graphs was observed. These results indicate that there are two dominant forms (G1 and G2) of VP1 unique domains which are structurally distinct from each other and different from those found in the outsider AAV isolates. This indicates that the genetic mosaic feature is indeed phenotypically expressed at the protein level.

Following the identification of two dominant forms of VP1 unique (R1) region, we searched for potential distinction of biological properties between G1 and G2 members. It was noted that G2 members analysed in Fig 3 are all of human origin, whilst G1 included isolates of both human and non-human primate (NHP) origin. Since this suggested that G1 and G2 might have different host ranges, the sample origin of all available primate AAV sequences were verified in their original reports. These sequences were classified into G1 (clustered with AAV1), G2 (clustered with AAV2) and others by making a phylogenetic tree for R1 (not shown) and summarised in Table 1. The majority of human AAV (50 sequences) belong to either AAV2-like clade B or AAV2/3 hybrid clade C which have been reported to be frequently found in the human population [10], [11]. These sequences have the G2 form of R1 (Table 1). In contrast the majority of NHP AAV contain the G1 form of R1 and no NHP AAV has the G2 form (Table 1). There are a substantial number (11 sequences) of human AAV with the G1 form (Table 1). These sequences clustered together with NHP AAV sequences in a mixed fashion along the whole cap gene (see Fig 2 and Fig3B). These results suggest that the G1 form of R1 is widely prevalent in primates and that G1 members of human origin may represent multiple cases of recent cross-species infection. Among the sequences classified as others, four sequences, hu 31, hu 32, hu 44 and hu 46, are apparent recombinant sequences identified in this study (Table 1). It is noteworthy that these sequences are of human origin and appear to have adopted G2 like forms of R1 (Fig 1B, 1C, Fig 2 R1 tree).

**Table 1:** Host distribution of G1/G2 forms.

R1 Group*	Human	NHP
G1	11	57
G2	50	0
Other	9 (4**)	2

* Number of primate AAV sequences in database were classified into G1, G2 and others according to R1 phylogeny.

** Apparent recombinants, hu 44/46 and hu 31/32, have G2-like form of R1

## Discussion

Our screening for recombinant sequences within 156 AAV *cap* sequences newly identified evidence for five recombination events. These events appear to be relatively recent recombination where the recombinant sequence and at least one parent sequence are closely related (Fig 2). This result, along with previous reports on AAV recombination [10]–[13] suggests that homologous recombination of AAV genomes occurs frequently. While the molecular mechanism of AAV recombination is still unclear, AAV has a number of properties that may facilitate recombination. In the presence of helper virus or upon genotoxic stress, the genome is efficiently converted into double-stranded DNA and amplified at high copy numbers [15], [16]. In addition, the inverted terminal repeats have a recombinogenic structure that can potentially attract cellular recombination and repair enzymes to the AAV genome [17]–[19]. Four out of the five crossover points identified here fall in and near the VP1 unique sequence, suggesting that recombination occurs frequently in this region. It is therefore likely that recombination has contributed to the mosaic structure of the *cap* gene within the AAV population in cases where the VP1 unique N-terminal segment and the C-terminal domain have different phylogenetic origins.

Two dominant forms of VP1 unique sequence were identified, whilst the remainder of the Cap protein has many diverse forms. The dominant AAV1-like form of VP1 unique region is prevalent in a wide range of primate AAV and linked to various forms of VP2/3 coding region (G1 sequences). The other dominant form represented by AAV2 is found in two previously reported lineages of human AAV, AAV2-like clade B represented by AAV2 and clade C represented by AAV2/3 hybrid isolates [10], [11]. The VP1 unique region encodes a phospholipase domain [7], which is enzymatically and structurally distinct from the VP3 capsomer unit. Our results suggest that dominant VP1 unique forms are restricted by enzymatic function and have been slowly evolving in their hosts. These forms could have become prevalent by convergent mutations and recombination purging less fit VP1 unique sequences. For example AAV11 and rh 32–34 sequences might have lost an unknown form of VP1 unique sequence by adopting AAV1 (also AAV10)-like form by recombination (Fig 1D and 2, R1 tree). In this context, frequent recombination around the VP2 initiation site (R1 and R2 junction) like those found in this study can contribute to the rise of dominant forms of VP1 unique region while enhancing diversification of the capsid structure domain. In other words, frequent, localised recombination within the *cap* gene may have resulted in conservation of one part and divergence in the other part, and in a genetic mosaic.

The idea that the dominant forms of VP1 unique/PLA domain have risen partly by recombination is consistent with the theory that protein building blocks are preserved by recombination [20]. The observed mosaicism within the AAV *cap* sequences suggests that recombination occurs around the junction of VP1 unique region more easily than in the middle of the *cap* domains. Indeed, artificial swapping of this region between AAV2 and AAV8 leads to a functional protein [21]. We therefore speculate that this junction may well be a recombination hot spot, representing a naturally occurring example of preservation of protein building blocks [20]. This will be interesting to consider in the scope of general evolutionary issues including the exon swapping theory [20], [22], [23]. Identification and swapping of a domain/region in artificial recombinant proteins may be useful for designing AAV vector systems. It will be interesting to study the behaviour of chimeric AAV *cap* genes where either of the two dominant forms of the VP1 unique domain are combined with various forms of capsid structural domains, in the context of both gene transfer vector and replicative infection systems.

The biological significance for the presence of two distinctive, dominant forms of VP1 unique sequence is unknown. One possibility is that this region affects AAV host range, as the AAV2-like form has been found exclusively in sequences derived from human samples whereas AAV1-like form has been found in both human and NHP samples (Table 1). These data however require cautious interpretation, because the sample number and range is limited. Notably most human isolates were characterized from samples collected in the Eastern part of the United States [10], [11], and therefore originate from a relatively small geographic area. However, the AAV2 epidemic in the human population may be global, as suggested by serological studies where it was found to be the prevalent type [24] and may be explained by a founder effect. Our finding that two lines of recombinant *cap* gene hu 31/32 and hu 44/46 have acquired AAV2 like VP1 unique sequence (Fig 1 and 2) may reflect a proliferation of this dominant form in human AAV population. It is also noteworthy that among NHP AAV sequences, a single sequence derived from chimpanzee, the ape species closest to humans has a VP1 unique sequence close to the AAV2-like form (Fig 2 R1 tree ch 5 sequence). Further investigation is warranted to test the possibility that this region affects the AAV host range. It is also possible that the distinctive VP1 unique forms coevolved with the upstream gene *rep*. Further sequencing of a wider range of AAV samples, ideally the whole genome including *rep* and host range studies on artificial AAV recombinants with the two dominant forms will test this hypothesis.

## Materials and Methods

### Retrieval and alignment of sequences

The capsid sequence of 156 AAV isolates derived from human, non-human primate and non-primate species were downloaded from GenBank (http://www.ncbi.nlm.nih.gov/). These sequences listed in **Table S1** were aligned using ClustalW [25] and manually curated to improve the quality of the alignment.

### Recombination detection and analyses

Recombination detection was performed initially on the entire 156 sequence dataset using a high throughput recombination detection method developed in-house. The Recombination Index (RecI) program used all possible triplets of sequences (***x, y, z***), determined the likelihood that one sequence was recombinant and that it was parented by the other two. The Rec program identified recombinants by scoring the percentage mismatch between sequence pairs using a sliding window. The question of whether sequence ***x*** was a recombinant between sequences ***y*** and ***z*** was answered by comparing ***x*** vs ***y*** and ***x*** vs ***z***. If sequence ***x*** was a recombinant, then sequence ***x*** would contain at least one region more like sequence ***y*** than sequence ***z*** and another region more like sequence ***z*** than sequence ***y***. These relationships were used to produce indices for all possible combinations of 3 sequences (recombinant/parents) from a multiple sequence data set. The details of the algorithm and the program executable are available on request.

The program LOHA [14] visualised conservation or divergence within and between sequence groups and was used to analyse potential recombination events indicated by the Rec analysis. LOHA was generated in-house and used a multiple alignment of sequences as its input. LOHA compared each of the possible pairs of aligned sequences with gaps and calculated the average percent mismatch across a sliding window of defined size (typically 41 Nt positions) and a step interval of 1 position. Positions with gaps for both sequences were counted as matched, while positions with a gap for only one sequence were counted as mismatched. The percent mismatch between a pair of sequences was plotted against the nucleotide positions using Microsoft Excel. This allowed regions of similarity or divergence to be visualised. Comparison of 3 LOHA plots (***x*** vs ***y***, ***x*** vs ***z***, ***y*** vs ***z)*** allowed possible recombination events between two parents and a progeny sequence to be determined. The output from a LOHA analysis of a multiple alignment was also grouped such that the average of the mismatch within a LOHA window was generated using all pairs of sequences input into the program. This output generated a single score per sliding window along the length of the alignment that was used to determine the level of nucleotide variation within a group of sequences (average group LOHA), as opposed to pairwise comparisons within the alignment (LOHA).

### Phylogenetic analyses

Phylogenetic analysis of AAV capsid sequences was performed to identify the evolutionary relationships between AAV strains, to identify clusters of highly related and therefore redundant sequences, and to verify the recombination events detected by the RecI and LOHA programs. Two methods of phylogenetic analysis were performed. The initial capsid phylogenies were generated using a bootstrapped (1000 replicates) neighbour-joining methodology (ClustalW). Trees for selected sequences were also generated using bootstrapped (1000 replicates) Phyml maximum likelihood trees [26] following estimation of an evolutionary model using Modeltest (Paup) [27] to confirm that this algorithm would generate similar phylogenies to those produced by a simpler evolutionary analysis. Bootstrapped neighbour-joining phylogenies were also performed on the partial alignments used to analyse recombinant breakpoints.

## Supporting Information

Table S1 List of 156 AAV cap gene sequences. A table to list up AAV cap gene sequenced used.
(0.15 MB DOC)

## References

[1] Hueffer K, Parrish CR (2003). Parvovirus host range, cell tropism and evolution.. Current Opinion in Microbiology.

[2] Shackelton LA, Parrish CR, Truyen U, Holmes EC (2005). High rate of viral evolution associated with the emergence of carnivore parvovirus.. PNAS.

[3] Wu Z, Asokan A, Samulski RJ (2006). Adeno-associated Virus Serotypes: Vector Toolkit for Human Gene Therapy.. Molecular Therapy.

[4] Xie Q, Bu W, Bhatia S, Hare J, Somasundaram T (2002). The atomic structure of adeno-associated virus (AAV-2), a vector for human gene therapy.. PNAS.

[5] Grieger JC, Johnson JS, Gurda-Whitaker B, Agbandje-McKenna M, Samulski RJ (2007). Surface-Exposed Adeno-Associated Virus Vp1-NLS Capsid Fusion Protein Rescues Infectivity of Noninfectious Wild-Type Vp2/Vp3 and Vp3-Only Capsids but Not That of Fivefold Pore Mutant Virions.. J Virol.

[6] Kronenberg S, Bottcher B, von der Lieth CW, Bleker S, Kleinschmidt JA (2005). A Conformational Change in the Adeno-Associated Virus Type 2 Capsid Leads to the Exposure of Hidden VP1 N Termini.. J Virol.

[7] Sonntag F, Bleker S, Leuchs B, Fischer R, Kleinschmidt JA (2006). Adeno-Associated Virus Type 2 Capsids with Externalized VP1/VP2 Trafficking Domains Are Generated prior to Passage through the Cytoplasm and Are Maintained until Uncoating Occurs in the Nucleus.. J Virol.

[8] Grieger JC, Snowdy S, Samulski RJ (2006). Separate Basic Region Motifs within the Adeno-Associated Virus Capsid Proteins Are Essential for Infectivity and Assembly.. J Virol.

[9] Zadori Z, Szelei J, Lacoste M-C, Li Y, Gariepy S (2001). A Viral Phospholipase A2 Is Required for Parvovirus Infectivity.. Developmental Cell.

[10] Gao G, Vandenberghe LH, Alvira MR, Lu Y, Calcedo R (2004). Clades of Adeno-Associated Viruses Are Widely Disseminated in Human Tissues.. J Virol.

[11] Chen C-L, Jensen RL, Schnepp BC, Connell MJ, Shell R (2005). Molecular Characterization of Adeno-Associated Viruses Infecting Children.. J Virol.

[12] Gao G, Alvira MR, Somanathan S, Lu Y, Vandenberghe LH (2003). Adeno-associated viruses undergo substantial evolution in primates during natural infections.. PNAS.

[13] Xiao W, Chirmule N, Berta SC, McCullough B, Gao G (1999). Gene Therapy Vectors Based on Adeno-Associated Virus Type 1.. J Virol.

[14] Bartosch B, Stefanidis D, Myers R, Weiss R, Patience C (2004). Evidence and consequence of porcine endogenous retrovirus recombination.. J Virol.

[15] Yalkinoglu AO, Heilbronn R, Burkle A, Schlehofer JR, zur Hausen H (1988). DNA amplification of adeno-associated virus as a response to cellular genotoxic stress.. Cancer Res.

[16] Weitzman MD, Fisher KJ, Wilson JM (1996). Recruitment of wild-type and recombinant adeno-associated virus into adenovirus replication centers.. J Virol.

[17] Raj K, Ogston P, Beard P (2001). Virus-mediated killing of cells that lack p53 activity.. Nature.

[18] Yan Z, Zak R, Zhang Y, Engelhardt JF (2005). Inverted Terminal Repeat Sequences Are Important for Intermolecular Recombination and Circularization of Adeno-Associated Virus Genomes.. J Virol.

[19] Zentilin L, Marcello A, Giacca M (2001). Involvement of Cellular Double-Stranded DNA Break Binding Proteins in Processing of the Recombinant Adeno-Associated Virus Genome.. J Virol.

[20] Voigt CA, Martinez C, Wang ZG, Mayo SL, Arnold FH (2002). Protein building blocks preserved by recombination.. Nat Struct Biol.

[21] Kohlbrenner E, Aslanidi G, Nash K, Shklyaev S, Campbell-Thompson M (2005). Successful production of pseudotyped rAAV vectors using a modified baculovirus expression system.. Mol Ther.

[22] de Souza SJ, Long M, Schoenbach L, Roy SW, Gilbert W (1996). Intron positions correlate with module boundaries in ancient proteins.. Proc Natl Acad Sci U S A.

[23] Gilbert W, de Souza SJ, Long M (1997). Origin of genes.. Proc Natl Acad Sci U S A.

[24] Erles K, Sebökovà P, Schlehofer J (1999). Update on the prevalence of serum antibodies (IgG and IgM) to adeno-associated virus (AAV).. Journal of Medical Virology.

[25] Thompson JD, Higgins DG, Gibson TJ (1994). CLUSTAL W: improving the sensitivity of progressive multiple sequence alignment through sequence weighting, position-specific gap penalties and weight matrix choice.. Nucleic Acids Res.

[26] Guindon S, Gascuel O (2003). A simple, fast, and accurate algorithm to estimate large phylogenies by maximum likelihood.. Syst Biol.

[27] Posada D, Crandall KA (1998). MODELTEST: testing the model of DNA substitution.. Bioinformatics.

