# Supplementary material for: Recombination and Population Mosaic of a Multifunctional Viral Gene, Adeno-Associated Virus cap
Source: PLoS One. 2008 Feb 20;3(2):e1634. doi: 10.1371/journal.pone.0001634 (PMC2238796; doi:10.1371/journal.pone.0001634)
Supplement: Table S1 — List of 156 AAV cap gene sequences. A table to list up AAV cap gene sequenced used. (0.15 MB DOC) [file pone.0001634.s001.doc]

# Supporting data

Table S1 List of 156 AAV cap gene sequences.

| GenBank Accession | Name | Origin | Fig 2* | Fig 3 | R1 class** |
| --- | --- | --- | --- | --- | --- |
| 46487868 | hu48 | Human | Y |  | 1 |
| 46487858 | hu43 | Human | Y |  | 1 |
| 46487848 | hu39 | Human | Y | G1 | 1 |
| 46487888 | hu6 | Human |  |  | 1 |
| 46487810 | hu17 | Human | Y | G1 | 1 |
| 46487856 | hu42 | Human |  |  | 1 |
| 46487852 | hu40 | Human |  |  | 1 |
| 46487900 | hu67 | Human | Y |  | 1 |
| 46487898 | hu66 | Human |  |  | 1 |
| 46487854 | hu41 | Human |  |  | 1 |
| 46487846 | hu37 | Human | Y |  | 1 |
| 46487808 | hu16 | Human |  |  | 2 |
| 46487806 | hu15 | Human |  |  | 2 |
| 46487812 | hu18 | Human |  |  | 2 |
| 46487902 | hu7 | Human |  |  | 2 |
| 46487892 | hu61 | Human |  |  | 2 |
| 46487890 | hu60 | Human | Y | G2 | 2 |
| 46487796 | hu1 | Human | Y | G2 | 2 |
| 46487816 | hu2 | Human |  |  | 2 |
| 46487850 | hu4 | Human |  |  | 2 |
| 46487836 | hu3 | Human |  |  | 2 |
| 46487828 | hu25 | Human |  |  | 2 |
| 46487800 | hu11 | Human |  |  | 2 |
| 46487904 | hu9 | Human |  |  | 2 |
| 46487798 | hu10 | Human |  |  | 2 |
| 51512247 | huS17 | Human | Y | G2 | 2 |
| 46487880 | hu55 | Human |  |  | 2 |
| 46487876 | hu53 | Human | Y | G2 | 2 |
| 46487878 | hu54 | Human |  |  | 2 |
| 46487870 | hu49 | Human |  |  | 2 |
| 46487886 | hu58 | Human |  |  | 2 |
| 46487802 | hu13 | Human |  |  | 2 |
| 46487834 | hu29 | Human |  |  | 2 |
| 46487832 | hu28 | Human |  |  | 2 |
| 46487844 | hu35 | Human |  |  | 2 |
| 46487842 | hu34 | Human |  |  | 2 |
| 46487862 | hu45 | Human |  |  | 2 |
| 46487866 | hu47 | Human |  |  | 2 |
| 46487872 | hu51 | Human |  |  | 2 |
| 46487874 | hu52 | Human |  |  | 2 |
| 2906016 | AAV2 | Human |  |  | 2 |
| 209616 | AAV2 | Human | Y | G2 | 2 |
| 51512238 | huT70 | Human | Y | G2 | 2 |
| 46487882 | hu56 | Human |  |  | 2 |
| 46487884 | hu57 | Human |  |  | 2 |
| 46487894 | hu63 | Human |  |  | 2 |
| 46487896 | hu64 | Human |  |  | 2 |
| 51512252 | huT41 | Human |  |  | 2 |
| 51512241 | huT71 | Human | Y | G2 | 2 |
| 51512230 | huT17 | Human |  |  | 2 |
| 51512232 | huT32 | Human | Y | G2 | 2 |
| 51512250 | huLG15 | Human |  |  | 2 |
| 51512235 | huT40 | Human | Y | G2 | 2 |
| 46487826 | hu24 | Human |  |  | 2 |
| 46487820 | hu21 | Human |  |  | 2 |
| 46487822 | hu22 | Human |  |  | 2 |
| 46487814 | hu19 | Human |  |  | 2 |
| 46487818 | hu20 | Human |  |  | 2 |
| 46487830 | hu27 | Human |  |  | 2 |
| 46487824 | hu23 | Human |  |  | 2 |
| 51512244 | huT88 | Human | Y | G2 | 2 |
| 4160146 | AAV5 | Human |  |  | O |
| 4249656 | AAV5 | Human | Y |  | O |
| 46487860 | hu44 | Human | Y |  | O |
| 46487864 | hu46 | Human | Y |  | O |
| 2766608 | AAV3_1 | Human | Y |  | O |
| 1408467 | AAV3_2 | Human | Y | O | O |
| 46487840 | hu32 | Human | Y |  | O |
| 46487838 | hu31 | Human | Y |  | O |
| 46487804 | AAV9 | Human | Y | O | O |
| 29650537 | ch5 | Chimpanzee | Y | O | O |
| 29650539 | bb2 | Baboon |  |  | 1 |
| 29650541 | bb1 | Baboon |  |  | 1 |
| 48728344 | AAV11 | Cynomolgus Macaque | Y |  | 1 |
| 48728341 | AAV10 | Cynomolgus Macaque | Y | G1 | 1 |
| 29650535 | cy2 | Cynomolgus Macaque | Y |  | 1 |
| 29650529 | cy5 | Cynomolgus Macaque |  |  | 1 |
| 29650533 | cy3 | Cynomolgus Macaque |  |  | 1 |
| 29650527 | cy6 | Cynomolgus Macaque | Y | G1 | 1 |
| 29650531 | cy4 | Cynomolgus Macaque |  |  | 1 |
| 46487752 | pi1 | Pigtailed macaque | Y | G1 | 1 |
| 46487756 | pi3 | Pigtailed macaque |  |  | 1 |
| 46487754 | pi2 | Pigtailed macaque |  |  | 1 |
| 2766605 | AAV6 | Primate | Y | G1 | 1 |
| 2337938 | AAV4 | Primate | Y | O | O |
| 29650501 | rh32 | Rhesus Macaque | Y |  | 1 |
| 29650499 | rh33 | Rhesus Macaque | Y |  | 1 |
| 29650497 | rh34 | Rhesus Macaque | Y |  | 1 |
| 4689096 | AAV1 | Rhesus Macaque | Y | G1 | 1 |
| 46487778 | rh53 | Rhesus Macaque |  |  | 1 |
| 46487774 | rh51 | Rhesus Macaque |  |  | 1 |
| 46487794 | rh64 | Rhesus Macaque |  |  | 1 |
| 46487772 | rh50 | Rhesus Macaque |  |  | 1 |
| 46487776 | rh52 | Rhesus Macaque |  |  | 1 |
| 46487790 | rh61 | Rhesus Macaque |  |  | 1 |
| 46487770 | rh49 | Rhesus Macaque | Y |  | 1 |
| 46487784 | rh57 | Rhesus Macaque |  |  | 1 |
| 46487786 | rh58 | Rhesus Macaque |  |  | 1 |
| 29650517 | rh16 | Rhesus Macaque |  |  | 1 |
| 29650515 | rh17 | Rhesus Macaque |  |  | 1 |
| 29650513 | rh18 | Rhesus Macaque |  |  | 1 |
| 29650523 | rh12 | Rhesus Macaque |  |  | 1 |
| 29650519 | rh14 | Rhesus Macaque |  |  | 1 |
| 29650503 | rh24 | Rhesus Macaque |  |  | 1 |
| 46487760 | rh25 | Rhesus Macaque |  |  | 1 |
| 29650525 | rh10 | Rhesus Macaque |  |  | 1 |
| 46487762 | rh38 | Rhesus Macaque | Y |  | 1 |
| 46487764 | rh40 | Rhesus Macaque |  |  | 1 |
| 29650509 | rh2 | Rhesus Macaque | Y | G1 | 1 |
| 46487766 | rh43 | Rhesus Macaque | Y |  | 1 |
| 22652862 | AAV8 | Rhesus Macaque | Y | G1 | 1 |
| 22652859 | AAV7 | Rhesus Macaque | Y | G1 | 1 |
| 46487780 | rh54 | Rhesus Macaque | Y | G1 | 1 |
| 29692329 | rh31 | Rhesus Macaque |  |  | 1 |
| 29692331 | rh26 | Rhesus Macaque |  |  | 1 |
| 29692333 | rh27 | Rhesus Macaque |  |  | 1 |
| 46487768 | rh48 | Rhesus Macaque | Y | G1 | 1 |
| 46487792 | rh62 | Rhesus Macaque |  |  | 1 |
| 46487782 | rh55 | Rhesus Macaque |  |  | 1 |
| 46487788 | rh60 | Rhesus Macaque | Y |  | 1 |
| 29650521 | rh13 | Rhesus Macaque |  |  | 1 |
| 29650507 | rh22 | Rhesus Macaque |  |  | 1 |
| 29650511 | rh19 | Rhesus Macaque |  |  | 1 |
| 29650505 | rh23 | Rhesus Macaque |  |  | 1 |
| 29650491 | rh37 | Rhesus Macaque |  |  | 1 |
| 29650493 | rh36 | Rhesus Macaque |  |  | 1 |
| 29650495 | rh35 | Rhesus Macaque | Y | G1 | 1 |
| 29650489 | rh8 | Rhesus Macaque | Y | G1 | 1 |
| 46487758 | rh1 | Rhesus Macaque | Y | G1 | 1 |
| 38679253 | BovineAAV | Bovine | Y |  |  |
| 85070096 | AAV-Go1 | Caprine |  |  |  |
| 52630844 | CaprineAAV1 | Caprine | Y |  |  |
| 73665994 | MouseAAV1 | Mouse | Y |  |  |
| 73665999 | RatAAV1 | Rat | Y |  |  |
| 38017148 |  | Snake |  |  |  |
| 48996102 | VR-865 | Avian |  |  |  |
| 31414777 | AvianAAV | Avian | Y |  |  |
| 48996105 | DA-1 | Avian |  |  |  |
| 38569533 | 90-0219 | Duck |  |  |  |
| 38569529 | 90-0219v | Duck |  |  |  |
| 38569535 | 97-0104 | Duck |  |  |  |
| 38569531 | 90-0215 | Duck |  |  |  |
| 609091 |  | Duck |  |  |  |
| 1113784 |  | Duck |  |  |  |
| 1134848 | 89384/FRANCE | Duck |  |  |  |
| 40846336 |  | Duck |  |  |  |
| 38569523 | 86-1015 | Goose |  |  |  |
| 38569515 | 82-0308 | Goose |  |  |  |
| 38569517 | 82-0321 | Goose |  |  |  |
| 38569527 | 01-1001 | Goose |  |  |  |
| 38569525 | 99-0808 | Goose |  |  |  |
| 38569519 | 82-0321v | Goose |  |  |  |
| 38569521 | 82-0408 | Goose |  |  |  |
| 73762558 | V325/TW03 | Goose |  |  |  |
| 1113795 | Virulent B | Goose |  |  |  |
| 984263 |  | Goose |  |  |  |

* Included in Fig 2; ** R1 Class: 1, G1; 2, G2; O, other
